# Influence of drought on plant performance through changes in belowground tritrophic interactions

**DOI:** 10.1002/ece3.4183

**Published:** 2018-06-25

**Authors:** Anouk Guyer, Bruce E. Hibbard, Annelie Holzkämper, Matthias Erb, Christelle A. M. Robert

**Affiliations:** ^1^ Institute of Plant Sciences University of Bern Bern Switzerland; ^2^ Oeschger Centre for Climate Change Research (OCCR) University of Bern Bern Switzerland; ^3^ Plant Genetics Research Unit USDA‐ARS University of Missouri Columbia Missouri; ^4^ Institute for Sustainability Sciences ISS Agroscope Zürich Switzerland

**Keywords:** climate change, *Diabrotica virgifera*, drought, multitrophic interactions, natural enemies, plant defense

## Abstract

Climate change is predicted to increase the risk of drought in many temperate agroecosystems. While the impact of drought on aboveground plant‐herbivore‐natural enemy interactions has been studied, little is known about its effects on belowground tritrophic interactions and root defense chemistry. We investigated the effects of low soil moisture on the interaction between maize, the western corn rootworm (WCR,* Diabrotica virgifera*), and soil‐borne natural enemies of WCR. In a manipulative field experiment, reduced soil moisture and WCR attack reduced plant performance and increased benzoxazinoid levels. The negative effects of WCR on cob dry weight and silk emergence were strongest at low moisture levels. Inoculation with entomopathogenic nematodes (EPNs, *Heterorhabditis bacteriophora*) was ineffective in controlling WCR, and the EPNs died rapidly in the warm and dry soil. However, ants of the species *Solenopsis molesta* invaded the experiment, were more abundant in WCR‐infested pots and predated WCR independently of soil moisture. Ant presence increased root and shoot biomass and was associated with attenuated moisture‐dependent effects of WCR on maize cob weight. Our study suggests that apart from directly reducing plant performance, drought can also increase the negative effects of root herbivores such as WCR. It furthermore identifies *S. molesta* as a natural enemy of WCR that can protect maize plants from the negative impact of herbivory under drought stress. Robust herbivore natural enemies may play an important role in buffering the impact of climate change on plant‐herbivore interactions.

## 1 INTRODUCTION

Root herbivores are among the most damaging pests in agriculture (Hunter, 2001). Their impact on plant performance depends on a number of factors, including host plant resistance (Huber et al., 2016), top‐down control by natural enemies (Degenhardt et al., 2009), and abiotic environmental conditions (Erb & Lu, 2013). In the context of climate change, temperature, soil moisture, and CO_2_ are increasingly recognized as abiotic modulators of root herbivore interactions (Hiltpold, Johnson, Bayon, & Nielsen, 2017; Johnson et al., 2010; McKenzie et al., 2016). Changes in soil moisture due to changes in rainfall patterns, including more frequent drought periods for instance, are predicted to profoundly change interactions between plants, root herbivores, and their natural enemies (Barnett & Facey, 2016; Hiltpold et al., 2017).

Soil moisture may influence belowground tritrophic interactions in several ways. First, it can directly affect the performance of plants (Chaves, Maroco, & Pereira, 2003), root herbivores (Barnett & Johnson, 2013; Johnson et al., 2010), and natural enemies (Grant & Villani, 2003; Rohde, Moino, Da Silva, Carvalho, & Ferreira, 2010). Second, soil moisture can alter the performance of root herbivores and their natural enemies indirectly via changes in plant chemistry (Khan, Ulrichs, & Mewis, 2010; Vaughan, Block, Christensen, Allen, & Schmelz, 2017). Third, root herbivores can alter the impact of soil moisture on plant performance, for instance by accentuating the negative impact of drought via the removal of roots by root herbivores (Erb et al., 2011).

**Figure 1:**
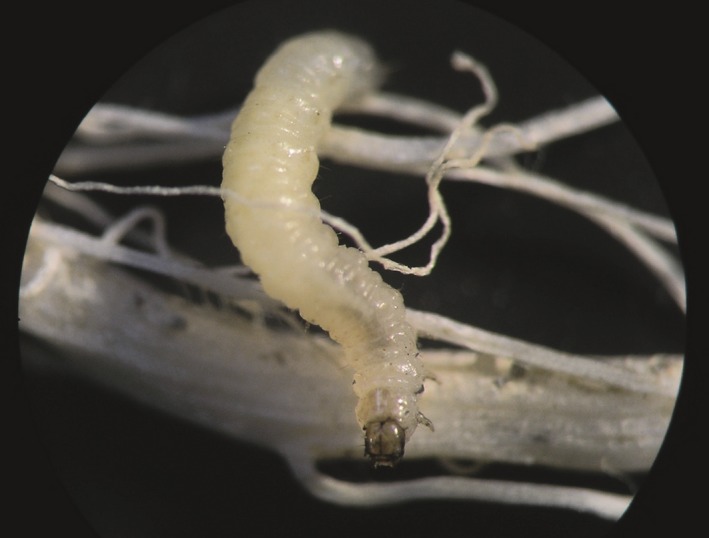
A first instar western corn rootworm larva (*Diabrotica virgifera virgifera*) attacking a maize root. Picture credit: Christelle A.M. Robert

Despite the fact that soil moisture effects on plants, root herbivores, and natural enemies have been described, little is known about how drought will modulate belowground tritrophic interactions and thereby influence plant performance. To address this question, we investigated the influence of soil moisture on the interaction between maize, the western corn rootworm (WCR, *Diabrotica virgifera virgifera*; Figure 1), and its natural enemies in a semifield experiment. WCR is among the most damaging maize pests and causes costs of up to 2 billion US$ per year in the United States alone (Mitchel, 2011). It is invasive in Europe (Miller et al., 2005; Wesseler & Fall, 2010), and current climate models predict that its range will expand as temperatures increase (Hemerik, Busstra, & Mols, 2004). Low soil moisture has been shown to reduce WCR survival and mobility at early developmental stages (Macdonald & Ellis, 1990; Spencer, Hibbard, Moeser, & Onstad, 2009). Conversely, the interactive effect of root herbivory by WCR and low soil moisture has been suggested to decrease maize performance in some cases (Mahmoud et al., 2016). Maize secondary metabolites, including benzoxazinoids (BXDs), and terpenoid phytoalexins such as zealexins and kauralexins, have been shown to increase in drought‐stressed maize plants (Erb et al., 2009; Vaughan et al., 2015), which in turn, may affect herbivore behavior and performance (Robert et al., 2012). Natural enemies of WCR include entomopathogenic nematodes (EPNs) as well as egg‐predatory mites, microbial pathogens or larval‐feeding ants (Krueger & Roberts, 1997; Kuhlmann & van der Burgt, 1998; Toepfer & Kuhlmann, 2004).

To study the influence of soil moisture on the maize WCR system, we planted maize plants in pots under a rainout shelter in Missouri (MO, USA) and varied soil water levels across a gradient from dry to intermediate soil moisture. We furthermore artificially applied EPNs and allowed for colonization of the pots by mobile natural enemies such as ants. We then measured root BXDs and plant performance as functions of soil moisture, WCR attack, and natural enemy presence. We hypothesized that drought reduces the capacity of natural enemies to control WCR and to alleviate its negative effects on plant performance.

## 2 MATERIAL AND METHODS

### 2.1 Plants, insects, and EPN

Maize seeds (Variety Quattro, DSP Delley, Switzerland) were sown in 9.9‐L plastic pots (Poly‐Tainer; Nursery Supplies, Minneville, OR, USA) filled with field soil (Hinkson Valley Farm, Boone Co., MO, USA). Drainage openings in each pot were covered with a fine (114 μm/opening) stainless steel mesh (TWP, Inc., Berkeley, CA, USA) to prevent larval escape. The field soil was comprised of 50% sand, 37.5% silt, and 12.5% clay (Clark et al., 2012). Pots were placed in the field (Hinkson Valley Farm, University of Missouri, Columbia, MO, USA) under a rainout shelter (steel construction with removable transparent plastic cover, PAK 1212C Clear Barrier, Hummert International, St. Louis, MO, USA). Maize plants were fertilized at the V4 and V7 stage with 300 ml of Peters Excel 15‐5‐15 Cal‐Mag (4 g/L). Nondiapausing eggs of *D*. *virgifera* (WCR) were obtained by mass rearing as described by Zukoff et al. (2016). *Galleria mellonella* larvae, which served as bait to test for EPN infectivity, were derived from Timberline (Timberline Fisheries, Marion, IL, USA). EPNs (*Heterorhabditis bacteriophora*) were bought from Koppert B.V (Larvanem^®^ from Koppert B.V., Howell, MI, USA).

### 2.2 Experimental design

One hundred and twenty‐one individual maize plants were sown and placed under the rainout shelter in a completely randomized design. Different soil moisture levels were established 51 days postsowing (V7 stage) and controlled by measuring soil water contents daily. Soil moisture was calculated by weighing the pots and subtracting their initial mass (soil dry weight [DW] and pot mass) as well as an estimation of the plant biomass (based on a correlation between plant size and total mass established under laboratory conditions, Supporting Information Figure S1). Plants were watered to establish a gradient of soil moisture ranging from 3.0% to 18.8% (Supporting Information Figure S2). The permanent wilting point was calculated at 12.1% gravimetric soil water content (Jabloun & Sahli, 2006). However, maize plants grew and developed even at the lowest soil moisture level. The addition of WCR and EPNs to the pots was performed following a full factorial design. Half of the maize plants (*n* = 60) were infested with 110 WCR eggs 28 days after planting (V4 stage) as described in Mahmoud et al. (2016). Larval hatching was monitored by controlling field‐incubated egg plates and infestation of a subset of pots. The hatching peak was reached 53 days after sowing. Separate incubation in a petri dish showed that 61.9% of the eggs were viable. Larval survival was measured by recovering larvae from three supplemental pots 60 days after planting. As no larvae were recovered from these pots, suggesting high WCR mortality, ten additional second to third instar WCR larvae were added to infested pots on day 63. Five thousand EPNs were added to half of control and half of WCR‐infested pots (*n* = 60) on day 64 after sowing (V11 stage) as described in Demarta, Hibbard, Bohn, and Hiltpold (2014). All plants were harvested at day 68. Final soil moisture levels were calculated based on final soil fresh mass and initial soil dry mass.

### 2.3 Plant phenotypic characterization

The anthesis‐silking interval (ASI) was calculated by counting the number of days between tassel development and silk appearance for each individual plant. Plant height was measured on day 64, as the distance from the soil surface to the base of the flag leaf. Shoot and cob FW were assessed on day 68. Shoot and root DW were assessed after plant harvest at day 68 and drying of the tissues in the greenhouse until weight loss was complete. All plants were phenotyped from 11:00 a.m. to 14:00 p.m.

### 2.4 WCR survival

Western corn rootworm survival was evaluated by suspending the plant roots and surrounding soil in mesh bags (New cabbage, USA) over water filled pans (6.65 L) in a greenhouse at day 68, causing the fall of soil‐dwelling invertebrates (Hibbard, Higdon, Duran, Schweikert, & Ellersieck, 2004). WCR larvae were counted twice a day for 12 days.

### 2.5 Thief ant identification and screening

Ants falling from the hanging mesh bags were collected on a fine mesh from water‐filled pans after 10 days and counted. Subsequently, the species was morphologically identified using the key to the genera of North American Myrmicinae developed by Fisher and Cover (2007).

### 2.6 EPN screening

Four soil cores of a diameter of 2.2 and 20 cm depth per pot were collected (day 67) at a distance of 5 cm from the stem. The soil cores of one pot were manually homogenized and used for EPN screening. Viable cruiser nematodes were collected by a modified Baermann Funnel extraction (Baermannm, 1917). Briefly, 120 g of homogenized soil aliquots were placed into a coffee filter (Brew Rite^®^, Walmart) into a plastic cup filled with a layer of tap water. To check for EPN presence and infectivity, three *G. mellonella* larvae were placed in a plastic cup (clear plastic cup with lid, 75 ml; Amscan Inc., USA) with 40 g of homogenized soil aliquots. After 6 days, dead *G. mellonella* larvae were transferred to modified white traps and EPN infection rates were recorded. Naturally occurring predator arthropods that fell from the soil samples were counted and collected for identification.

### 2.7 Benzoxazinoid analysis

Three crown root sections (2–4 cm) were excised from all plants (day 65) and immediately frozen in liquid nitrogen for secondary metabolite analyses. Crown roots were ground to a fine powder in a mortar containing liquid nitrogen. An aliquot of 100 mg FW of roots was extracted by adding 1 ml of acidified H_2_O: MeOH (50:50 v/v; 0.5% formic acid). Analyses were performed on a FW rather than DW basis to determine actual metabolite concentrations encountered by herbivores and natural enemies. BXDs were analyzed using an Acquity UHPLC system coupled to a G2‐XS QTOF mass spectrometer equipped with an electrospray source (Waters, USA). The elution gradient was realized on an Acquity BEH C18 column (2.1 × 50 mm, 1.7 μm particle size) as follow: 99%–72.5% A over 3.5 min, 100% B over 2 min, holding at 99% A for 1 min, where A = 0.1% formic acid/water and B = 0.1% formic acid/acetonitrile. The flow rate was 0.4 ml/min. The column temperature was maintained at 40°C, and the injection volume was 1 μl. The QTOF MS was operated in negative mode. The data were acquired over an m/z range of 50–1,200 with scans of 0.15 s at collision energy of 4 V and 0.2 s with a collision energy ramp from 10 to 40 V. The capillary and cone voltages were set to 2 kV and 20 V, respectively. The source temperature was maintained at 140°C, the desolvation was 400°C at 1,000 L/hr and cone gas flows was 50 L/hr. Accurate mass measurements (<2 ppm) were obtained by infusing a solution of leucin encephalin at 200 ng/ml at a flow rate of 10 μl/min through the Lock Spray probe (Waters). BXDs were identified using synthetic standards and characteristic m/z fragments following Glauser et al. (2011).

### 2.8 Statistical analysis

The data were statistical analyzed in R (R Version 3.4.1) using R studio (Rstudio Version 1.0.143). The experiment followed a completely randomized three‐way factorial design, with WCR and ant presence as categorical variables and soil moisture as a continuous variable. Ant presence and absence assignment was based on the number of ants collected. Pots with more than 5 ants were classified as “ants present”, while pots with less than five ants were considered as “ants absent”. Because of incomplete soil removal, two samples were excluded for the root DW and root shoot ratio measurements. One sample was excluded from the cob data measurements because of incomplete drying. Response factors were analyzed using generalized linear models (glm), with soil moisture, WCR infestation and ant presence as fixed effects. EPN application was not included as an explanatory variable into the model, as no evidence for successful EPN establishment was found (see Results). The models contained all possible interactions between explanatory variables. Chi‐square tests were conducted to evaluate such interactions. All Chi‐square tests had a degree of freedom of 1. The models were tested for normality and equality of variance using the package RVAideMemoire (Hervé, 2016). Final models contained rank‐transformed soil moisture values. The response variables ant count, HBOA‐Glc and HM_2_BOA‐Glc were log‐transformed and cob weight was sqrt‐transformed, to fit normality and equality of variance. Effects on response variables were analyzed using ANOVA. Effects with *p*‐values below 0.05 were considered significant.

## 3 RESULTS

### 3.1 Survival and abundance of root herbivores and natural enemies

**Figure 2:**
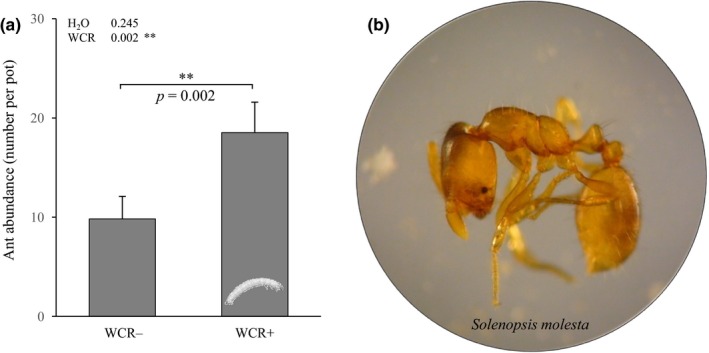
Influence of WCR infestation on the abundance of *Solenopsis molesta* ants. (a) Average number of *S. molesta* individuals (+SE) that are present in pots of WCR‐infested (*n* = 58) or WCR‐uninfested plants (*n* = 59). (b) Microscope picture of a *S. molesta* worker ant. *p*‐values of treatment effects are indicated (*p ≤ 0.05, **p ≤ 0.01, ***p ≤ 0.001)

A total of 15 WCR larvae were recovered from WCR‐infested pots, indicating low infestation levels (Table 1). No WCR were found in uninfested pots. Approximately 150 nematodes were extracted per 100 g of soil DW (Supporting Information Figure S3). Soil moisture and WCR presence did not influence cruiser nematode density (Supporting Information Figure S4). Also, EPN addition did not significantly change the abundance of total nematodes (Supporting Information Figure S3). *G. mellonella* larvae placed in soil aliquots did not yield to any infection by EPNs within 6 days, indicating that the added EPNs did not establish successfully in EPN+ pots and that biological control through native EPNs in control pots was negligible. The thief ant, *Solenopsis molesta*, was identified as the most abundant, naturally present, predator in the pots. Field observations revealed that these ants attacked WCR larvae (Supporting Information Figure S5a). The thief ants were more abundant in WCR‐infested pots (glm: χ^2^_WCR_ = 20.921, p_WCR_ = 0.002; ANOVA_WCR_: F = 10.401, *p* = 0.002; Figure 2a), independently from soil moisture and pot position in the field plot (Supporting Information Figure S5b).

**Table 1:** WCR recovery at the end of the experiment

Treatment	Number of pots with recovery (total number of pots)	Total number of recovered WCR
Control	0 (35)	0
WCR	6 (21)	13
Ants	0 (23)	0
Ants+WCR	2 (38)	2

Note

The number of pots from which WCR were recovered as well as the total number of recovered WCR per treatment are shown. Total number of pots per treatment are given in brackets.

### 3.2 Influence of soil moisture on plant performance

**Figure 3:**
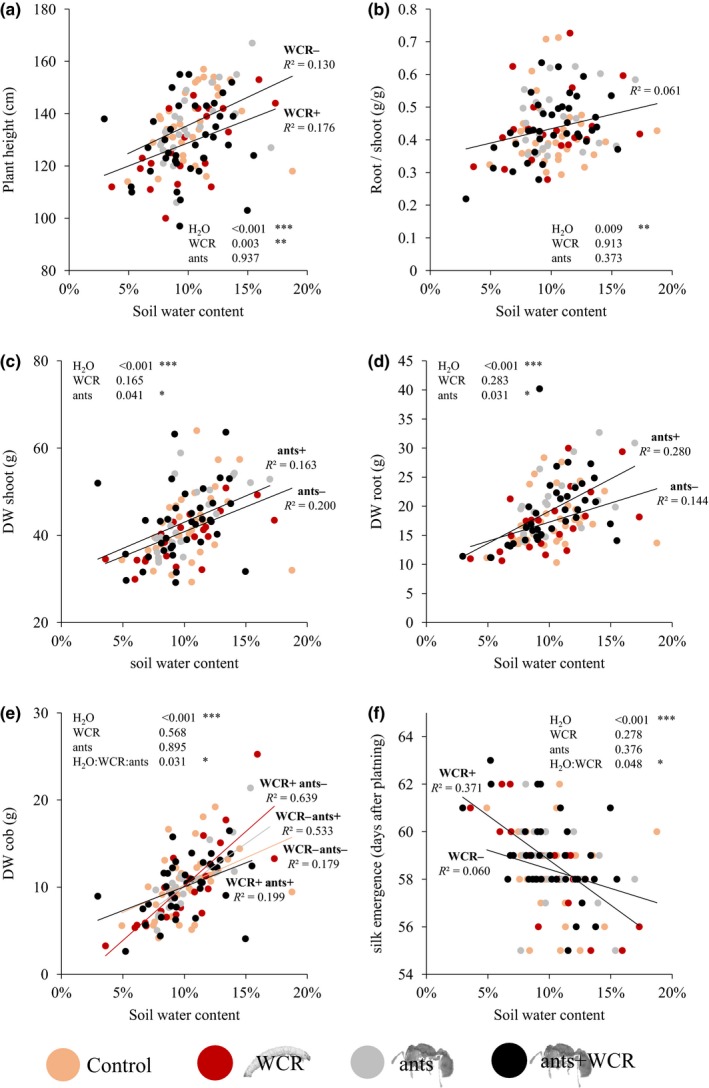
Maize performance varies with soil moisture, WCR infestation and *Solenopsis molesta* presence. Dots represent individual plants separated into four treatments: Controls (orange, *n* = 35), WCR‐infested plants (red, *n* = 21), plants with *S. molesta* (gray, *n* = 23), and WCR‐infested plants with *S. molesta* (black, *n* = 38). Soil water contents are indicated in % on the *x*‐axis. Significant treatment and interaction effects are visualized by linear regression lines, including *R* ^2^‐values. (a) Plant height (in cm), (b) Ratio between root and shoot dry weights, (c, d) Shoot and root dry weights, (e) Cob dry weight and (f) Time to silk emergence (in days after planting). *p*‐values of treatment effects and significant interactions are indicated (*p ≤ 0.05, **p ≤ 0.01, ***p ≤ 0.001)

Depending on the daily temperature and light intensity, maize plants showed wilting symptoms, especially at noon and at low soil moisture levels. Nevertheless, all plants grew continuously, produced tassels and silks as well as one to two cobs (Figure 3). Reduced soil moisture resulted in proportionally smaller plants (glm: χ^2^_H2O_ = 28.656, p_H2O_ < 0.001) with lower shoot weight (glm: χ^2^_H2O_ = 35.297, p_H2O_ < 0.001), root weight (glm: χ^2^_H2O_ = 44.615, p_H2O_ < 0.001) and lower root/shoot ratios (glm: χ^2^_H2O_ = 6.773, p_H2O_ = 0.009). Cob weight was also significantly reduced by low soil moisture (glm: χ^2^_H2O_ = 75.888, p_H2O_ < 0.001). Time to silk emergence (glm: χ^2^_H2O_ = 29.338, p_H2O_ < 0.001) and ASI (glm: χ^2^_H2O_ = 35.018, p_H2O_ < 0.001) on the other hand were increased at lower moisture levels (Figure 3 and Supporting Information Figure S6).

### 3.3 Interactive effects of root herbivory and soil moisture on plant performance

WCR infestation significantly reduced plant height (glm: χ^2^_WCR_ = 9.017, p_WCR_ = 0.003; Figure 3a), but did not influence root or shoot DW as well as root/shoot ratios (Figure 3b). We also detected a significant interaction between WCR infestation and silk emergence (glm: χ^2^_H2O:WCR_ = 3.917, p_H2O:WCR_ = 0.048; Figure 3f): At low moisture, WCR infestation increased the time to silk emergence, while it decreased time to silk emergence at higher moisture levels compared to non‐infested plants (Figure 3f). Cob DW was also influenced by WCR in interaction with soil moisture and natural enemy presence, as discussed below (Figure 3e).

### 3.4 Interactive effects of soil moisture, herbivory and natural enemies on plant performance

Most likely due to the low survival and infectivity as well as the relatively short time interval between EPN application and plant harvest, EPN addition did not have any impact on WCR‐dependent plant performance (data not shown). *S. molesta* presence, on the other hand, was associated with increased root and shoot DW (Figure 3c,d). We also detected a significant three‐way interaction between soil moisture, WCR infestation and *S. molesta* presence on cob DW (glm: χ^2^_H2O:WCR:ants_ = 4.630, p_H2O:WCR:ants_ = 0.031; Figure 3e). Visual inspection of linear regressions (Figure 3e) indicated that WCR infestation decreased cob weight at low soil moisture, but increased it at high soil moisture compared to non‐infested controls. In the presence of ants, both the positive and negative effects of WCR on cob DW were absent.

### 3.5 Influence of soil moisture and herbivory on root secondary metabolites

**Figure 4:**
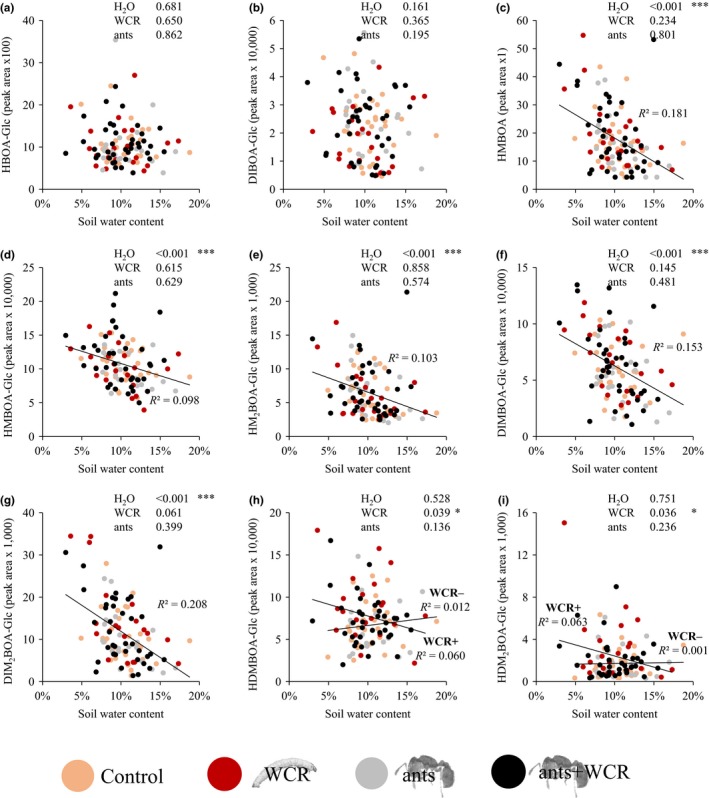
Root benzoxazinoid levels are influenced by soil moisture and WCR infestation. Relative amounts of benzoxazinoids in crown roots of individual plants are shown. Controls (orange, *n* = 30), WCR‐infested plants (red, *n* = 21), plants with *Solenopsis molesta* (gray, *n* = 21), and WCR‐infested plants with *S. molesta* (black, *n* = 38). Soil water contents are indicated in % on the *x*‐axis. (a) HBOA‐Glc, (b) DIBOA‐Glc, (c) HMBOA, (d) HMBOA‐Glc, (e) HM_2_ BOA‐Glc, (f) DIMBOA‐Glc, (g) DIM_2_ BOA‐Glc, (h) HDMBOA‐Glc, (i) HDM_2_ BOA‐Glc. Significant treatment and interaction effects are visualized by linear regression lines, including *R* ^2^‐values. *p*‐values of treatment effects and significant interactions are indicated (*p ≤ 0.05, **p ≤ 0.01, ***p ≤ 0.001)

Low soil moisture significantly and linearly increased the levels of several BXDs, including HMBOA, HMBOA‐Glc, HM_2_BOA‐Glc, DIMBOA‐Glc and DIM_2_BOA‐Glc (glm: χ^2^_H2O_ > 14.900, p_H2O_ < 0.001). Relative abundances were two‐ to threefold higher at low soil moisture compared to high soil water contents (Figure 4). WCR infestation increased the concentrations of HDMBOA‐Glc (glm: χ^2^_WCR_ = 4.252, p_WCR_ = 0.039; Figure 4h) and HDM_2_BOA‐Glc (glm: χ^2^_WCR_ = 4.394, p_WCR_ = 0.036, Figure 4i). We did not detect any significant interactions between WCR infestation and soil moisture.

### 3.6 Interactive effects of soil moisture, herbivory and natural enemies on plant performance

Ant presence was not correlated with root benzoxazinoid levels. We also did not detect any significant interactions between ant presence, WCR infestation and/or soil moisture (Figure 4).

## 4 DISCUSSION

This study found that soil moisture altered the impact of a root herbivore on maize performance, and that these changes were absent in pots that were colonized by a naturally occurring herbivore enemy. Plant chemistry, on the other hand, was influenced by soil moisture and herbivory, but not related to the presence of natural enemies. Below, we discuss the implications of these findings in an agroecological context.

Drought alone reduced plant performance, but increased the production of root secondary metabolites. The reduction in plant biomass, height, ASI and cob mass observed in this study is consistent with previous findings (Barnabás, Jäger, & Fehér, 2008; Witt et al., 2012). ASI and cob weight are strong indicators of plant yield and are used as a trait to select for drought‐resistant maize cultivars (Cattivelli et al., 2008). Unexpectedly and in contrast to other studies (Vaughan et al., 2015), we found that low moisture decreased root/shoot ratios. This result may be explained by the relatively severe drought regime, with plants showing clear stress symptoms at lower soil moisture levels. Severe drought stress reduces maize root biomass directly (Aslam, Maqbool, & Cengiz, 2015) and inhibits compensatory root growth (Hansen, Hauggaard‐Nielsen, Petersen, Mikkelsen, & Müller‐Stöver, 2016), which may result in decreased root/shoot ratios.

The substantially increased levels of BXDs in maize roots under low soil moisture are in accordance with earlier results showing an increase in 2,4‐dihydroxy‐7‐methoxy‐2H‐1,4‐benzoxazin‐3‐one (DIMBOA) and 2,4‐dihydroxy‐3H‐1,4‐benzoxazin‐3‐one (DIBOA) in drought‐stressed maize seedlings (Richardson & Bacon, 1993). Maize roots have also been found to accumulate terpenoid phytoalexins under drought stress, which may increase plant resistance to water deficit (Vaughan et al., 2015). Whether BXDs fulfill a similar role remains to be investigated. In addition, to what extent the increased concentrations are the result of reduced root water contents is currently unclear. As BXDs have an important role in plant defense and protection against herbivores and pathogens (Ahmad et al., 2011; Glauser et al., 2011; Maag et al., 2016), the drought‐induced concentration increase may also alter plant interactions with other plant‐associated organisms (Erb et al., 2011; Robert et al., 2012).

Soil moisture may influence the impact of herbivores on plant performance (Gray & Steffey, 1998; Jamieson, Trowbridge, Raffa, & Lindroth, 2012) and chemistry (Erb & Lu, 2013; Nguyen, Rieu, Mariani, & van Dam, 2016). We found that WCR attack reduced plant height and accentuated the effect of low soil moisture on the onset of silk emergence. Silks are the maize tissues with the highest water content and respond strongly to soil water deficit (Aslam et al., 2015; Fuad‐Hassan, Tardieu, & Turc, 2008). The fact that WCR attack further accentuated the delay in silk emergence points to a further increase in drought stress in WCR‐attacked plants. Small maize seedlings were shown previously to suffer from increased leaf water loss upon combined drought and WCR attack (Erb et al., 2011). Potential interactive effects between water deficit and WCR attack have also been noted episodically in the field (Godfrey, Meinke, & Wright, 1993; Urías‐López, Meinke, Higley, & Haile, 2000). A detailed greenhouse study, however, did not find any significant interactive effects between WCR and soil moisture on vegetative growth and water potentials (Mahmoud et al., 2016). Compensatory root growth may have allowed plants to tolerate WCR under drought conditions in this case (Robert et al., 2014). Our data shows that WCR effects can become visible in the generative stage even if biomass accumulation is not changed. These late effects may reflect costs of the plant resistance and/or tolerance strategies, as resources used for secondary metabolite production and compensatory growth would be diverted from the development of reproductive organs.

Western corn rootworm attack increased HDMBOA‐Glc and HDM_2_BOA‐Glc levels in the roots. These two BXDs are well documented to be induced in the leaves of herbivore‐attacked maize plants and are known to be potent defense metabolites against herbivores (Glauser et al., 2011). WCR, however, is tolerant to BXDs (Robert et al., 2012) and is therefore unlikely to be affected by the increased levels in induced plants. Whether the induction of HDMBOA‐Glc and HDM_2_BOA‐Glc leads to cross‐resistance against other root feeders remains to be investigated.

Inoculation with EPNs did not affect the interaction between maize, WCR, and soil moisture, as the EPNs died rapidly in the warm and dry soil. As EPNs are highly susceptible to desiccation (Kung, Gaugler, & Kaya, 1990; Pilz et al., 2012), their efficacy is likely to decrease rapidly with climate change. The water gradient in our experiment ranged from very low to low moisture levels, and it is likely that the soil conditions were unsuitable for the added EPNs, even at higher moisture levels. On the other hands, the thief ant *S. molesta* naturally colonized WCR‐infested plants in the field independently of soil moisture levels and was repeatedly observed to predate WCR. *Solenopsis molesta* is widespread in the Nearctic region and can be found in different habitats and soil types such as grasslands or arid sites (Pacheco, Mackay, & Lattke, 2013). *Solenopsis molesta* is omnivorous and can feed on other ants, insects and insect eggs (Rao & Vinson, 2009; Vinson & Rao, 2004; Zenger & Gibb, 2001) as well as plant seeds (Pacheco et al., 2013). Root biomass was increased in the presence of ants, suggesting a positive influence of their presence on plant performance. While the induction of root secondary metabolites was not associated with ant presence, we found a correlation between ant presence and the soil moisture‐dependent impact of WCR on cob weight: Regression analysis showed that WCR reduced cob weight under low soil moisture, but tended to increase it under high soil moisture. If ants were present, both effects were no longer observed. This result suggests that the presence of ants effectively reduces the soil moisture‐dependent impact of WCR on this important plant performance parameter, and can therefore buffer the impact of drought on plant‐herbivore interactions.

Interactions between soil moisture, plants, herbivores and natural enemies are expected to depend on a number of factors, including (a) the range of soil moisture levels (Aslam, Johnson, & Karley, 2013; Mahmoud et al., 2016; Schmitz & Barton, 2014), (b) the timing of changes in soil moisture relative to the development of all three trophic levels (Rosenblatt & Schmitz, 2016; Schmitz & Barton, 2014; Wade, Karley, Johnson, Hartley, & Bell, 2017) (c) the plant genotype (Stam et al., 2014), (d) the severity of herbivore attack (Soler, Bezemer, van der Putten, Vet, & Harvey, 2005) and (e) the abundance and diversity of herbivore natural enemies (Erb & Lu, 2013; Thomson, Macfadyen, & Hoffmann, 2010). Our study was conducted at low soil moisture close to the plant's wilting point, relatively low WCR infestation levels and a relatively short period between the application of EPNs and plant performance measurements. Further experiments will therefore be needed to understand whether the observed patterns represent general properties of belowground tritrophic interactions.

This work suggests that *S. molesta* may be an effective biological control agent for WCR, but the potential and off‐target effects of this species require further investigation. Overall, this study shows that robust natural enemies can alleviate the negative interactive effects of herbivory and drought on plant performance. Identifying herbivore natural enemies which maintain their biocontrol potential under variable abiotic conditions can help buffering the impact of climate change on plant‐herbivore interactions**.**

## CONFLICT OF INTEREST

None declared.

## AUTHOR CONTRIBUTIONS

AG designed and carried out the field experiment, analyzed data, and wrote the manuscript. BEH assisted in the design and provided the infrastructure for the field experiment. AH contributed to the design of the study, analyzed data, and wrote the manuscript. ME designed the study and the field experiment, analyzed data, and wrote the manuscript. CAMR designed the study and the field experiment, analyzed data, and wrote the manuscript.

## Supporting information


** **
