# Supplementary material for: Influence of drought on plant performance through changes in belowground tritrophic interactions
Source: Ecol Evol. 2018 Jun 25;8(13):6756–65. doi: 10.1002/ece3.4183 (PMC6053580; doi:10.1002/ece3.4183)
Supplement: Supplementary file 1 [file ECE3-8-6756-s001.pptx]

## Slide 1
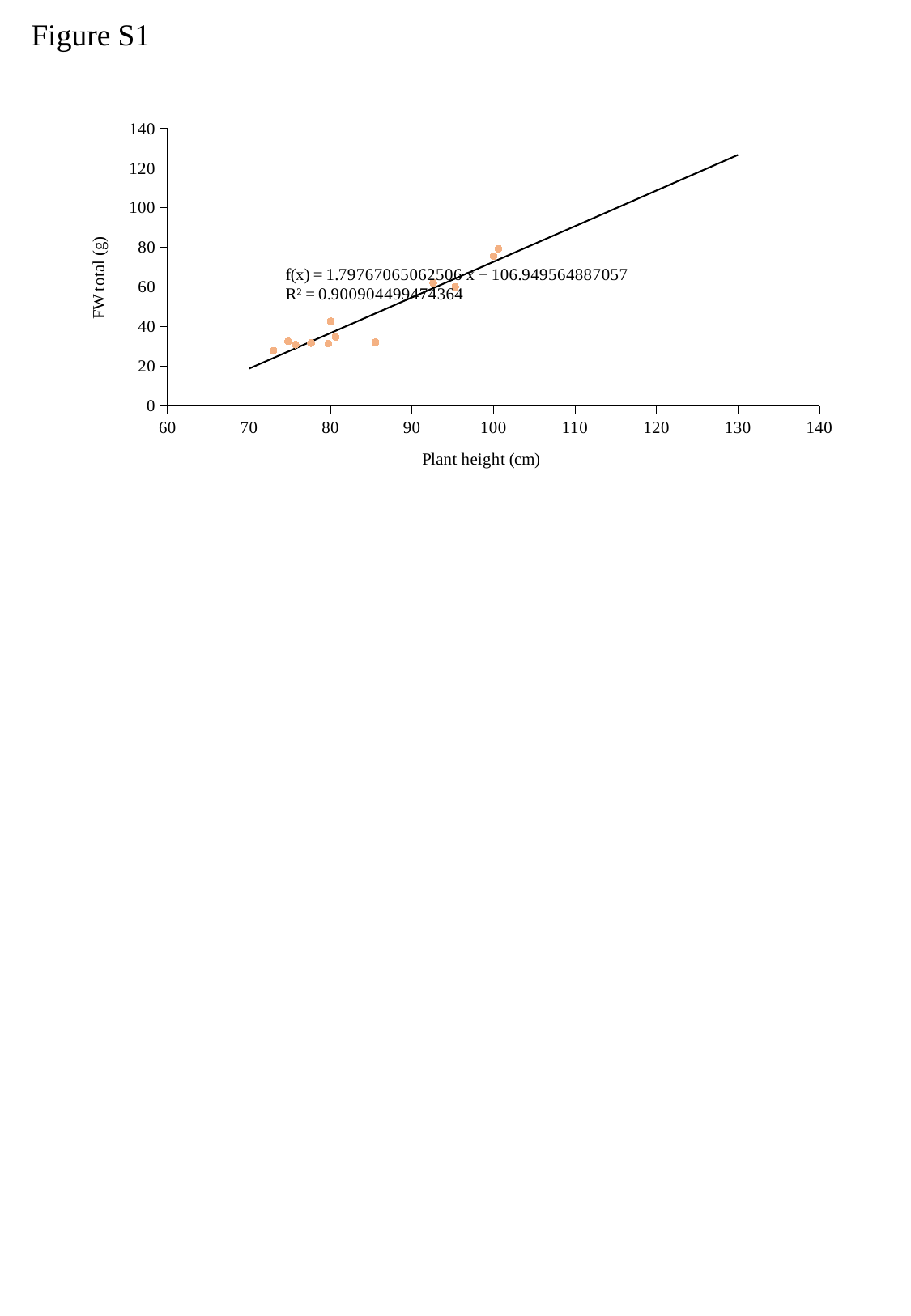

Figure S1
### Chart
| Category | total FW
[g] | |
|---|---|---|

## Slide 2
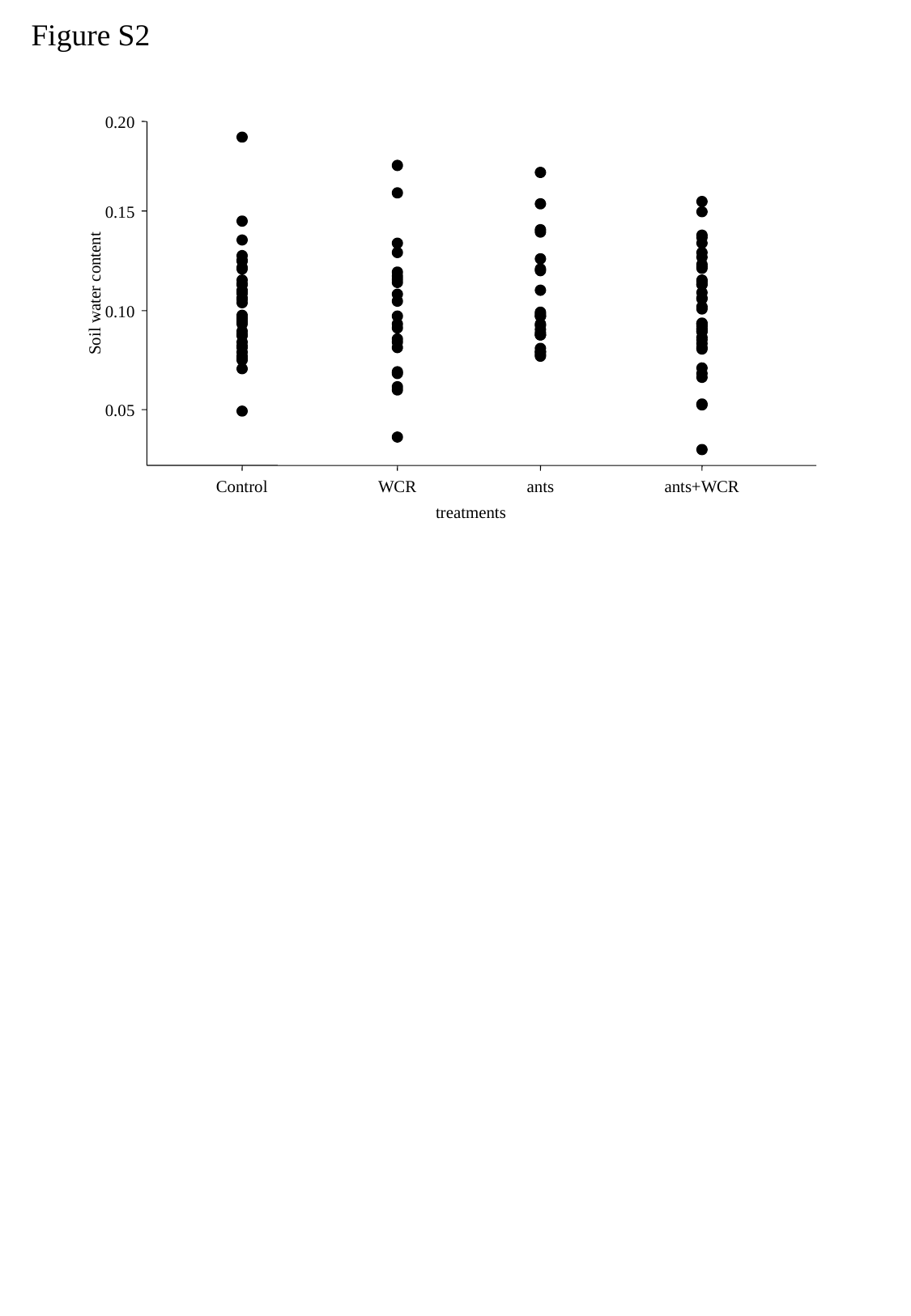

Figure S2
0.20
Control
WCR
ants
ants+WCR
0.15
Soil water content
0.10
0.05
treatments

## Slide 3
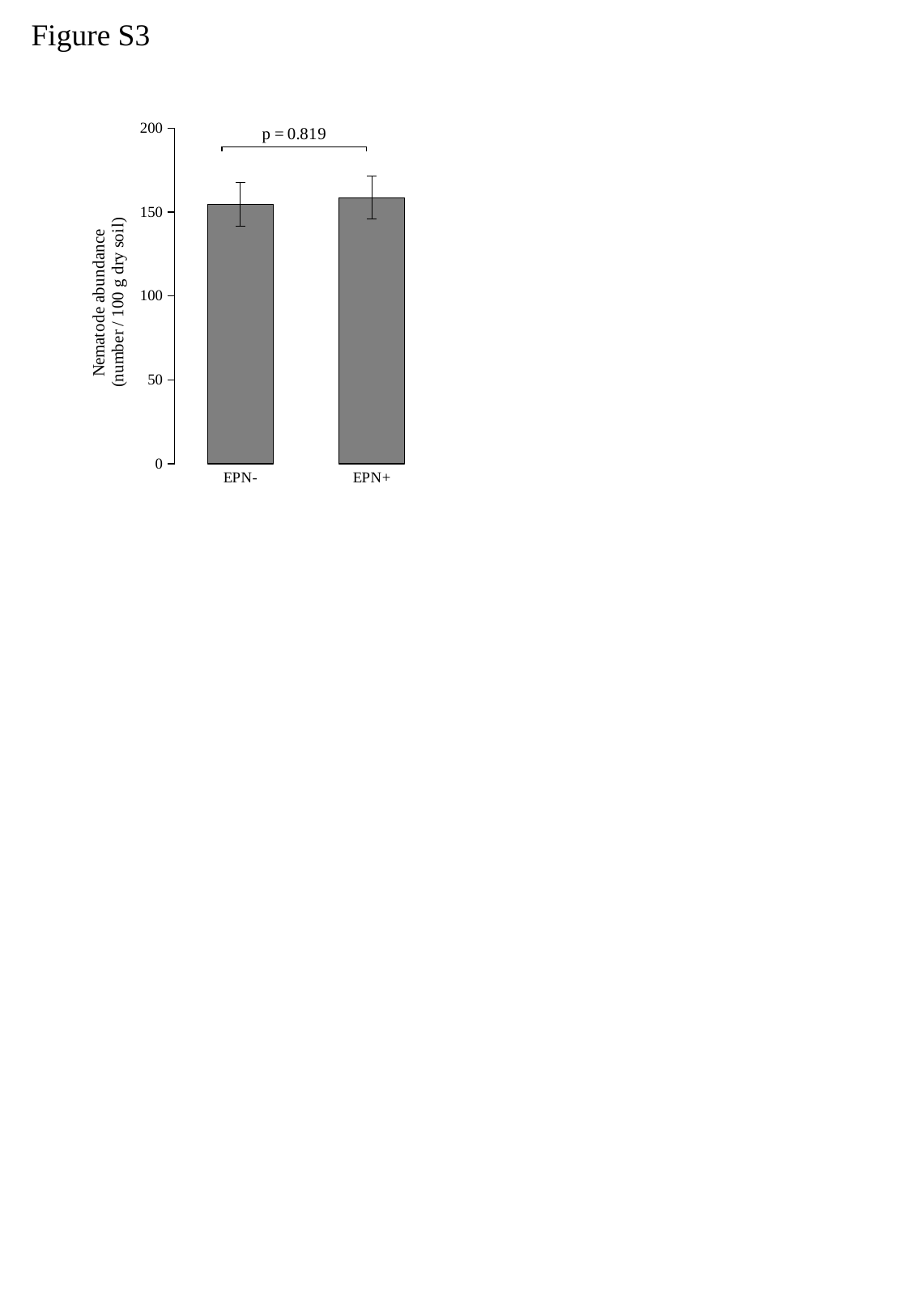

Figure S3
### Chart
| Category | |
|---|---|
| EPN- | 154.39617322393977 |
| EPN+ | 158.57683276463345 |

## Slide 4
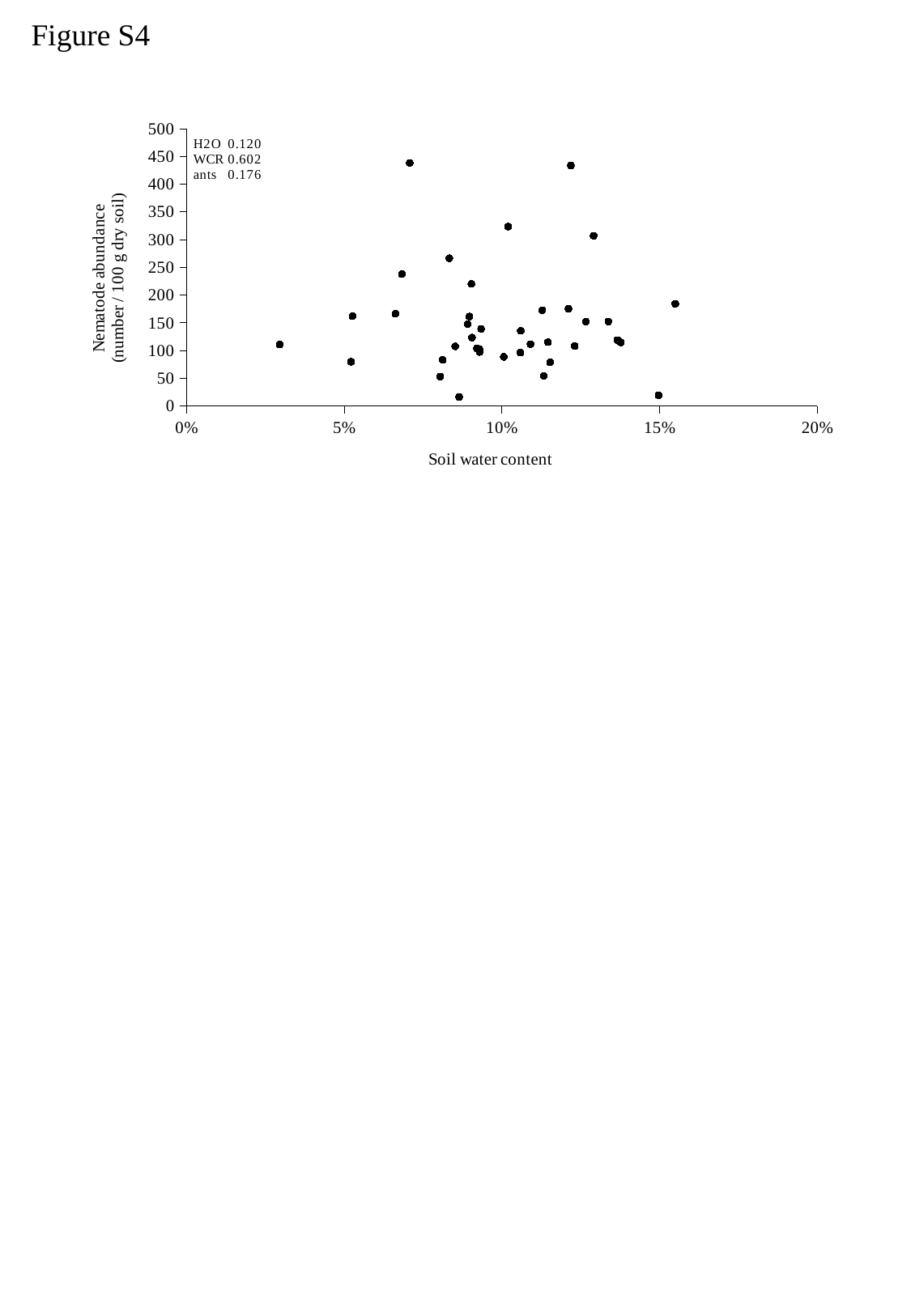

Figure S4
### Chart
| Category | | | | |
|---|---|---|---|---|

## Slide 5
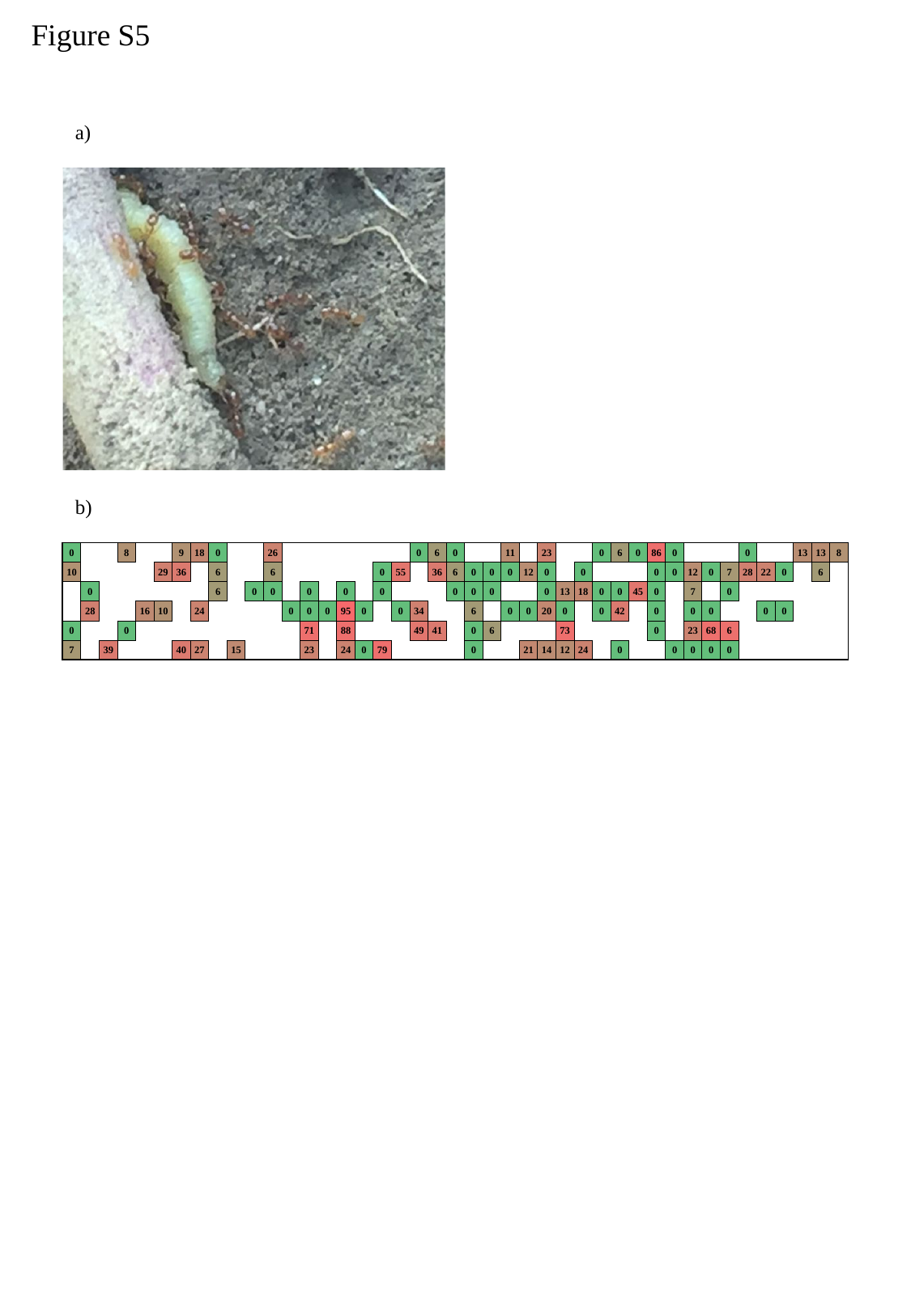

Figure S5
a)
b)
| 0 | | | 8 | | | 9 | 18 | 0 | | | 26 | | | | | | | | 0 | 6 | 0 | | | 11 | | 23 | | | 0 | 6 | 0 | 86 | 0 | | | | 0 | | | 13 | 13 | 8 |
| --- | --- | --- | --- | --- | --- | --- | --- | --- | --- | --- | --- | --- | --- | --- | --- | --- | --- | --- | --- | --- | --- | --- | --- | --- | --- | --- | --- | --- | --- | --- | --- | --- | --- | --- | --- | --- | --- | --- | --- | --- | --- | --- |
| 10 | | | | | 29 | 36 | | 6 | | | 6 | | | | | | 0 | 55 | | 36 | 6 | 0 | 0 | 0 | 12 | 0 | | 0 | | | | 0 | 0 | 12 | 0 | 7 | 28 | 22 | 0 | | 6 | |
| | 0 | | | | | | | 6 | | 0 | 0 | | 0 | | 0 | | 0 | | | | 0 | 0 | 0 | | | 0 | 13 | 18 | 0 | 0 | 45 | 0 | | 7 | | 0 | | | | | | |
| | 28 | | | 16 | 10 | | 24 | | | | | 0 | 0 | 0 | 95 | 0 | | 0 | 34 | | | 6 | | 0 | 0 | 20 | 0 | | 0 | 42 | | 0 | | 0 | 0 | | | 0 | 0 | | | |
| 0 | | | 0 | | | | | | | | | | 71 | | 88 | | | | 49 | 41 | | 0 | 6 | | | | 73 | | | | | 0 | | 23 | 68 | 6 | | | | | | |
| 7 | | 39 | | | | 40 | 27 | | 15 | | | | 23 | | 24 | 0 | 79 | | | | | 0 | | | 21 | 14 | 12 | 24 | | 0 | | | 0 | 0 | 0 | 0 | | | | | | |

## Slide 6
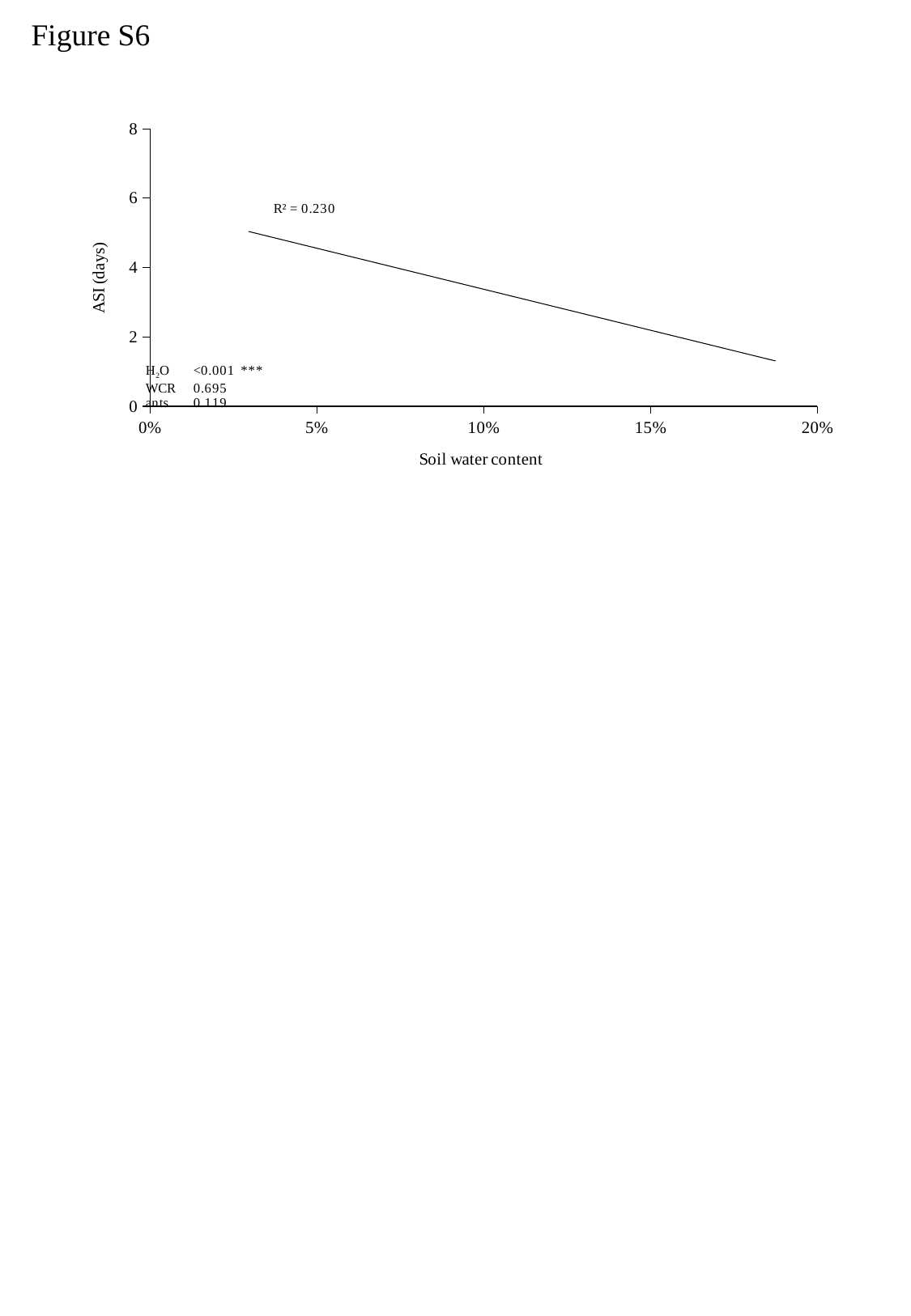

Figure S6
### Chart
| Category | WCR-.ant- | WCR+.ant- | WCR-.ant+ | WCR+.ant+ | |
|---|---|---|---|---|---|
